# First molecular evidence of bovine hemoplasma species (*Mycoplasma* spp.) in water buffalo and dairy cattle herds in Cuba

**DOI:** 10.1186/s13071-019-3325-y

**Published:** 2019-02-07

**Authors:** Adrian Alberto Díaz-Sánchez, Belkis Corona-González, Marina L. Meli, Dasiel Obregón Álvarez, Ernesto Vega Cañizares, Osvaldo Fonseca Rodríguez, Evelyn Lobo Rivero, Regina Hofmann-Lehmann

**Affiliations:** 1 Centro Nacional de Sanidad Agropecuaria (CENSA), Carretera de Tapaste y Autopista Nacional, Apartado postal 10, 32700 San José de las Lajas, Mayabeque Cuba; 2 Clinical Laboratory, Department of Clinical Diagnostics and Services, and Center for Clinical Studies, Vetsuisse Faculty, University of Zurich, Zurich, Switzerland; 3 Facultad de Medicina Veterinaria, Universidad Agraria de la Habana (UNAH), Apartado postal 18-19, 32700 San José de las Lajas, Mayabeque Cuba; 4 Department of Epidemiology and Global Health, Umeå University, Umeå, Sweden; 5 Centre for Demographic and Ageing Research, Umeå University, Umeå, Sweden

**Keywords:** Hemotropic mycoplasma, *Mycoplasma wenyonii*, “*Candidatus* Mycoplasma haemobos”, Real-time PCR, *16S* rRNA

## Abstract

**Background:**

Hemotropic mycoplasmas (aka hemoplasmas) are small bacteria which cause infectious anemia in several mammalian species including humans. Information on hemoplasma infections in Cuban bovines remains scarce and no studies applying molecular methods have been performed so far. The aim of the present study was to utilize real-time PCR and sequence analysis to investigate dairy cattle and buffalo from Cuba for the presence of bovine hemoplasma species.

**Results:**

A total of 80 blood samples from 39 buffalo and 41 dairy cattle were investigated for the presence of *Mycoplasma wenyonii* and “*Candidatus* Mycoplasma haemobos” using two species-specific real-time TaqMan PCR assays. PCR results revealed overall 53 (66.2%; 95% CI: 55.3–75.7%) positive animals for *M. wenyonii* and 33 (41.2%; 95% CI: 31.1–52.2%) for “*Ca.* M. haemobos”; the latter were all co-infections with *M. wenyonii*. The sample prevalences were similar in cattle and buffalo. Based on the sequence analysis of the nearly full-length *16S* rRNA gene from two cattle and two buffalo, the presence of *M. wenyonii* and “*Ca.* M. haemobos” was confirmed. Statistical analysis revealed that buffalo and cattle one year of age or older were more frequently infected with *M. wenyonii* or “*Ca.* M. haemobos” than younger animals. PCR-positivity was not associated with anemia; however, the infection stage was unknown (acute infection *versus* chronic carriers).

**Conclusions:**

The high occurrence of bovine hemoplasma infections in buffalo and dairy cattle may have a significant impact on Cuban livestock production. To the best of our knowledge, this is the first molecular evidence of bovine hemoplasma species infection in dairy cattle and buffalo from Cuba and the Caribbean.

## Background

Hemotropic mycoplasmas or hemoplasmas are small epierythrocytic gram-negative bacteria, which can only survive by parasitism of erythrocytes and cause infectious anemia in several mammalian species including humans [1, 2]. Hemoplasmas were originally classified as members of the two genera of order Rickettsiales, namely *Eperythrozoon* and *Haemobartonella*. Some years ago, these organisms have been reclassified as members of the genus *Mycoplasma* based on strong phylogenetic evidence of their *16S* rRNA sequences and morphological similarity [3, 4].

In cattle, two distinct hemotropic *Mycoplasma* species have been identified to date: *Mycoplasma wenyonii* (Mw: formerly *Eperythrozoon wenyonii*) [5] and a provisional species “*Candidatus* Mycoplasma haemobos” (CMh: synonym “*Candidatus* M. haemobovis”) [6, 7]. Clinical signs of hemoplasma infection in cattle include anemia, transient fever, lymphadenopathy, anorexia, weight loss and decreased milk production; in most animals, however, infection remains subclinical [8]. The epidemiology of bovine hemotropic mycoplasmas is still poorly understood, and the possible transmission routes may include by vectors such as fleas, hard ticks and mosquitoes, or by direct contact with contaminated blood [9, 10].

*Mycoplasma wenyonii* was first described by Adler et al. [5] in a splenectomized calf; since then it has been reported throughout the world [1]. In Cuba, *M. wenyonii* was first reported in cattle by Pino et al. [11]; however we cannot be certain that *M. wenyonii* was the species truly detected since molecular techniques were not available at the time. As part of the original report, splenectomized calves were inoculated with the agent and it was determined that anemia could be induced under experimental conditions [12]. Despite the first report occurring 30 years ago, the agent has since neither been reported in Cuba nor in any other Caribbean country [13]. The second bovine hemoplasma species, “*Ca.* M. haemobos” has been reported more recently for the first time using molecular methods such as polymerase chain reaction (PCR) and DNA sequencing techniques in cattle from several countries around the world, including the Americas mainland [6, 14–17].

Hemoplasmas have never been cultured *in vitro*. A tentative diagnosis may be based on cytological examination of erythrocytes on Giemsa stained blood smears by microscopy. However, this method has a low analytical sensitivity and specificity because the organism resembles Howell-Jolly bodies or background debris and other artifacts, and the bacterial loads are very low during chronic infection leaving detection by microscopy nearly impossible [18]. The development of PCR based methods led to assays with increased sensitivity and specificity for the identification of bovine hemoplasma species; these methods represent the useful diagnostic methods of choice nowadays [14, 19].

To the best of our knowledge, molecular detection of bovine hemoplasmas has never been reported to date in the Caribbean countries. Thus, the aim of the present study was to utilize molecular biological techniques, namely real-time PCR and sequence analysis, to investigate the presence of bovine hemoplasma species in cattle and buffalo from Cuba.

## Results

**Fig. 1:**
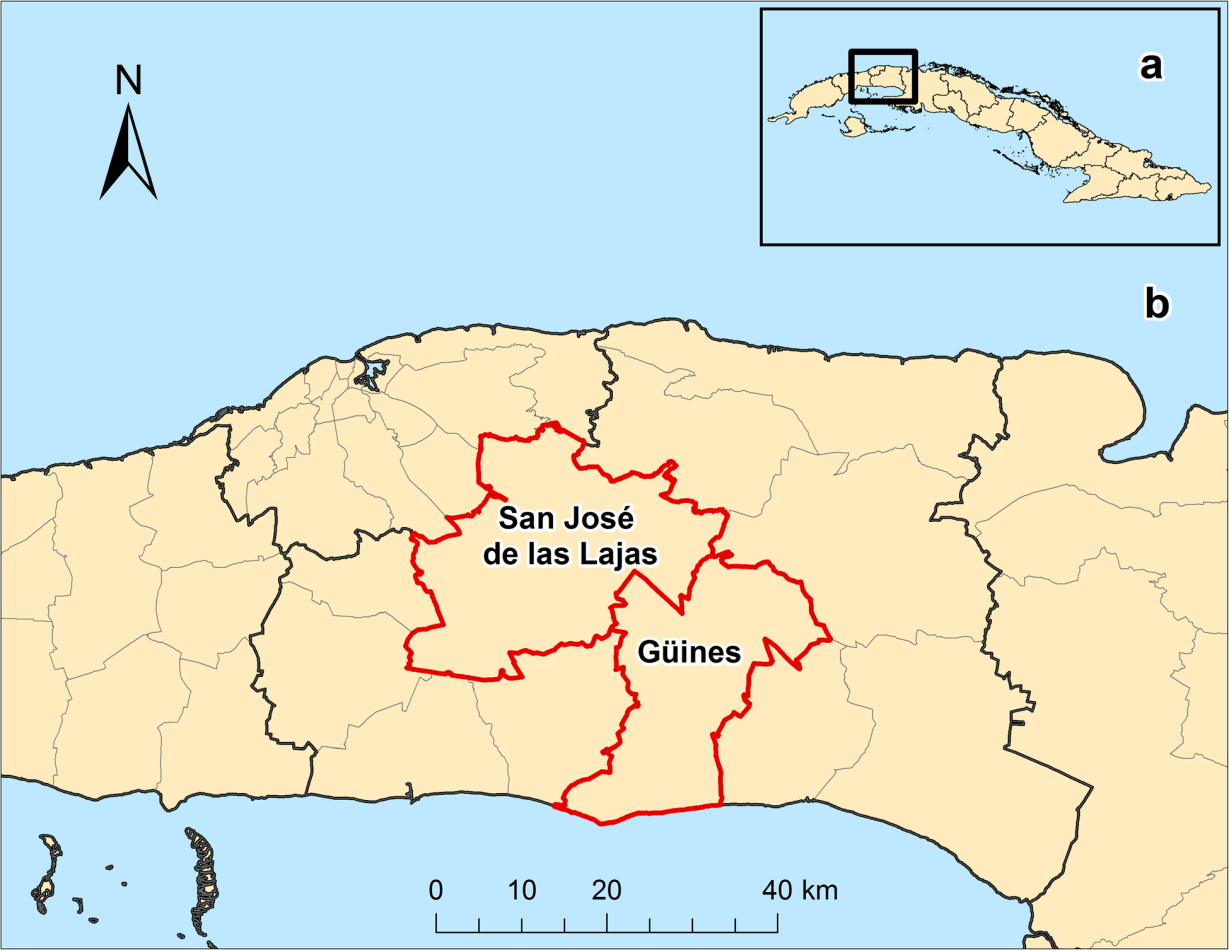
Map of the study area. **a** Island of Cuba. **b** Location of the farms where the sample collection was conducted in the municipalities of Güines and San José de las Lajas, Mayabeque Province. *Scale-bar*: 40 km

A total of 80 blood samples were collected from two cattle (*Bos taurus*) and two water buffalo (*Bubalus bubalis*) herds in the Mayabeque Province of Cuba (Fig. 1) and investigated for bovine hemoplasmas using molecular assays. Information concerning sex, age, tick infestation, management system and hematocrit of all animals was obtained (Table 1). In all 80 total nucleic acid (TNA) samples extracted from the blood samples, a sufficient amount of DNA was present as determined by GAPDH PCR (Ct value < 22). All extraction and non-template controls were PCR-negative; all positive controls tested PCR-positive. Overall, 53 of the 80 tested animals (66.2%; 95% CI: 55.3–75.7%) from either cattle or buffalo were PCR-positive for bovine hemoplasmas. All 53 PCR-positive animals were positive for *M. wenyonii* (overall sample prevalence of *M. wenyonii* 66.2%; 95% CI: 55.3–75.7%) and 33 of 80 were positive for “*Ca.* M. haemobos” (overall sample prevalence of “*Ca.* M. haemobos” 41.2%; 95% CI: 31.1–52.2%). Among the PCR-positive animals, 33 were co-infected with *M. wenyonii* and “*Ca.* M. haemobos”; 18 tested positive for *M. wenyonii* only and none of these animals was positive only for “*Ca.* M. haemobos”.

**Table 1:** Sample characteristics

Variable	No. of animals
Species	
Cattle	41
Buffalo	39
Sex	
Female	74
Male	6
Age	
< 1 year	41
1–3 years	39
Tick infestation	
Yes	44
No	36
Management system	
Semi-extensive	41
Extensive	39

Among the 41 dairy cattle, 26 (63.4%; 95% CI: 48.0–76.4%) and 18 (43.9%; 95% CI: 29.8–59.0%) animals showed PCR-positive results for *M. wenyonii* and “*Ca.* M. haemobos”, respectively. Co-infections were detected in 18 of 41 (43.9%; 95% CI: 29.8–59.0%) cattle blood samples analyzed, 8 tested positive for *M. wenyonii* only and none were positive only for “*Ca.* M. haemobos”. Out of 39 buffalo, 27 (69.2%; 95% CI: 53.5–81.4%) and 15 (38.5%; 95% CI: 24.9–54.2%) animals showed PCR-positive results for *M. wenyonii* and “*Ca.* M. haemobos”, respectively. Co-infections were detected in 15 of 39 (38.5%; 95% CI: 24.9–54.2%) buffalo blood samples analyzed, 12 tested positive for *M. wenyonii* only and none were positive only for “*Ca.* M. haemobos”. There was no significant difference in the total hemoplasma prevalence or in the percentage of single or dual positive samples in cattle compared to buffalo.

The hemoplasma-infected cattle and buffalo did not exhibit any clinical signs attributable to hemoplasmosis, such as pale mucous membranes, transient fever, lymphadenopathy, anorexia, weight loss or decreased milk production. No statistically significant differences in packed cell volume (PCV) values were found between infected (range 19–42%, average 34.07 ± 5.02) and uninfected (range 27–45%, average 37.08 ± 4.40) buffalo (Mann Whitney U-test, *U* = 299, *P* = 0.072), as well as infected (range 23–36%, average 28.65 ± 3.68) and uninfected (range 20–42%, average 28.67 ± 6.88) cattle (Mann Whitney U-test, *U* = 297.5, *P* = 0.634). Moreover, infected buffalo and cattle were not more frequently anemic than non-infected animals (Tables 2, 3). Animals that were one-year-old or older were significantly more frequently infected with *M. wenyonii* or “*Ca.* M. haemobos” compared to animals less than one year of age (Fisher’s exact test, *P* < 0.05); this was the case for both cattle and buffalo (for details see Tables 2, 3). There was no association between sex of the animals and bovine hemoplasma species prevalence (data not shown). All studied animals originated from pastureland; the dairy cattle herds were maintained under a semi-intensive management system, while the buffalo herds were reared under an extensive system.

**Table 2:** Distribution of variables identified with *M. wenyonii* and “*Ca*. M. haemobos” infections in buffalo herds in Mayabeque Province, Cuba

Variable	qPCR *M. wenyonii*		OR	95% CI	*P-*value	qPCR “*Ca.* M. haemobos”		OR	95% CI	*P-*value
	Positive (*n* = 27)	Negative (*n* = 12)				Positive (*n* = 15)	Negative (*n* = 24)			
Hematocrit										
< 30	4 (15%)	1 (9%)	1.913	0.25–25.3	>0.9999	2 (13%)	3 (13%)	1.08	0.17–5.85	>0.9999
30–50	23 (85%)	11 (91%)				13 (87%)	21 (88%)			
Age										
< 1 year	9 (33%)	11 (91%)	0.045	0.004–0.3	**0.0012**	0 (0%)	20 (83%)	0	0–0.077	**<0.0001**
1–3 years	18 (67%)	1 (9%)				15 (100%)	4 (17%)			
Ticks										
No	18 (67%)	4 (33%)	4.0	0.89–14.0	0.082	15 (100%)	7 (29%)	∞	7.48–∞	**<0.0001**
Yes	9 (33%)	8 (67%)				0 (0%)	17 (71%)			

*Abbreviations*: OR, odds ratio; 95% CI, 95% confidence interval

Significant *P*-values (Fisher’s exact test: *P* < 0.05) are indicated in bold

**Table 3:** Distribution of variables identified with *M. wenyonii* and “*Ca*. M. haemobos” infections in dairy cattle herds in Mayabeque Province, Cuba

Variable	qPCR *M. wenyonii*		OR	95% CI	*P-*value	qPCR “*Ca.* M. haemobos”		OR	95% CI	*P-*value
	Positive (*n* = 26)	Negative (*n* = 15)				Positive (*n* = 18)	Negative (*n* = 23)			
Hematocrit										
< 24	1 (4%)	4 (27%)	0.11	0.009–0.86	0.0514	1 (6%)	4 (17%)	0.42	0.02–2.08	0.3629
24–47	25 (96%)	11 (73%)				17 (94%)	19 (83%)			
Age										
< 1 year	9 (35%)	12 (80%)	0.13	0.035–0.58	**0.0088**	5 (28%)	16 (70%)	0.17	0.04–0.62	**0.0122**
1–3 years	17 (65%)	3 (20%)				13 (72%)	7 (30%)			
Ticks										
No	11 (42%)	3 (20%)	2.93	0.67–11.3	0.1860	9 (50%)	15 (65%)	0.53	0.14–1.82	0.3583
Yes	15 (58%)	12 (80%)				9 (50%)	8 (35%)			

*Abbreviations*: OR, odds ratio; 95% CI, 95% confidence interval

Significant *P*-values (Fisher’s exact test: *P* < 0.05) are indicated in bold

All ticks collected from dairy cattle and buffalo were identified as *Rhipicephalus microplus*, which is known as the cattle tick. A total of 129 (92 females and 37 males) and 148 (96 females and 52 males) adult ticks were collected from cattle and buffalo, respectively. Parasitism by at least one tick was detected in 27 of 41 (65.9%; 95% CI: 49.4–79.9%) cattle and 17 of 39 (43.6%; 95% CI: 27.8–60.4%) buffalo. An indirect association between “*Ca.* M. haemobos” infection prevalence and tick-infestation was observed in buffalo only: buffalo without tick infestation were significantly more frequently infected with “*Ca.* M. haemobos” than buffalo with ticks (Fisher’s exact test, *P <* 0.0001; for details see Table 2).

Identification of hemoplasma species was further demonstrated based on sequence analysis of the nearly full-length *16S* rRNA gene sequence of four hemoplasma-positive samples (two buffalo samples; two cow samples). Two sequences designated as BovMw31 and BufMw03 (GenBank: MG948624 and MG948626, respectively) were 99% identical to each other and exhibited > 99% identity with *M. wenyonii* strain Massachusetts (GenBank: CP003703). While the two other sequences designated BovCMhbos61 and BufCMhbos01 (GenBank: MG948628 and MG948631, respectively) were 99% identical to each other and exhibited > 99% identity with those of “*Ca.* M. haemobos” clones 307 and 311 derived from infected cows in Switzerland (GenBank: EF616467 and EF616468, respectively).

**Fig. 2:**
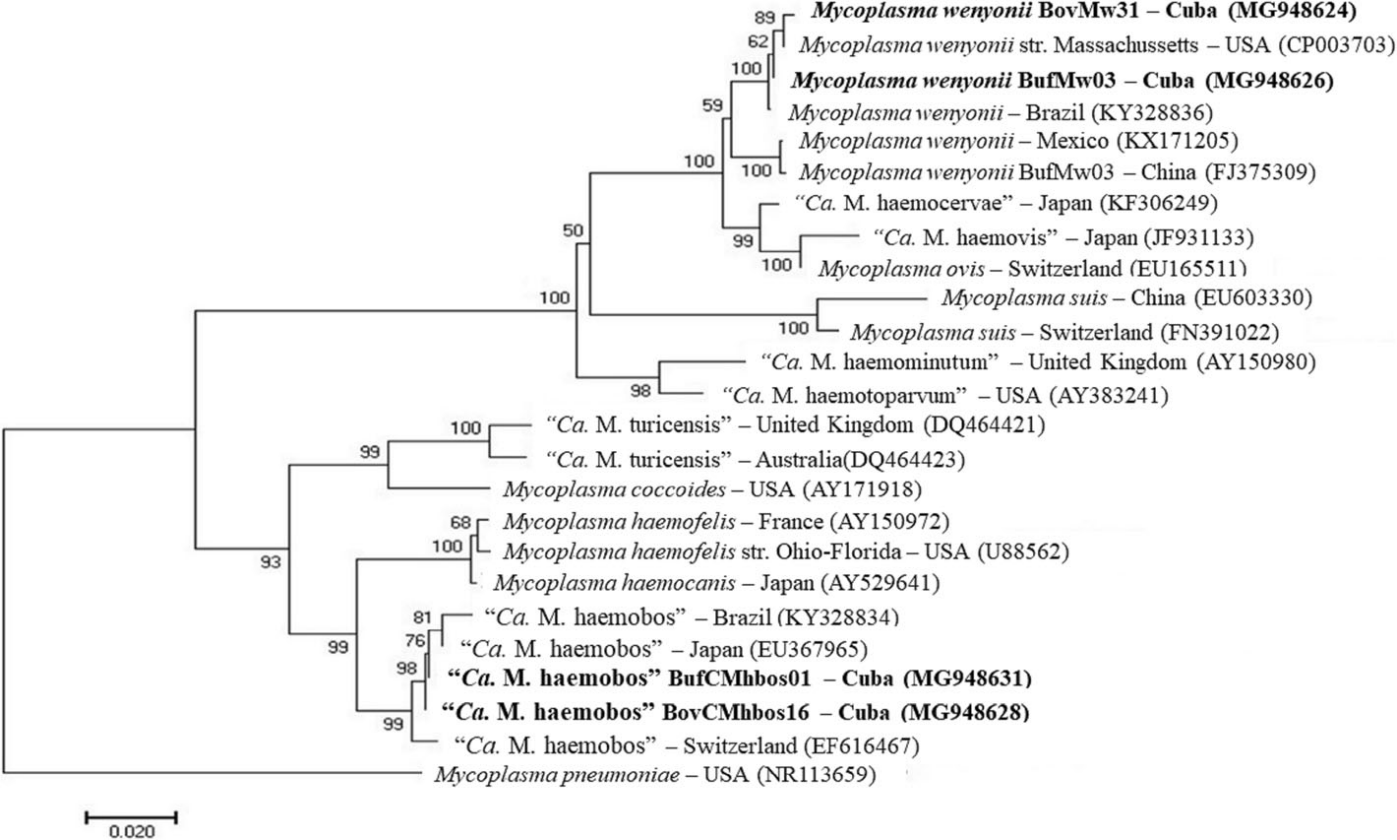
Phylogenetic analysis of *M. wenyonii* and “*Ca*. M. haemobos” isolates from dairy cattle and buffalo. The analysis was based on a nearly-full length 16S rRNA gene sequence comparison. The phylogenetic tree was constructed using the maximum likelihood (ML) method based on the Tamura 3-parameter model, and the numbers above the internal nodes indicate the percentages of 1000 bootstrap replicates that supported the branch. *Mycoplasma pneumoniae* (NR113659) was used as outgroup. GenBank accession numbers are shown in parentheses. The *M. wenyonii* (BovMw31, BufMw03) and “*Ca*. M. haemobos” (BovCMhbos61, BufCMhbos01) samples identified in the present study are indicated in bold

A phylogenetic tree based on the nearly full-length *16S* rRNA gene sequences confirmed the close evolutionary relationship between the *M. wenyonii* Cuban isolates (BovMw31 and BufMw03) with other isolates from China and the USA (Fig. 2), and clustered into the clade of *M. wenyonii*. The Cuban isolates of “*Ca.* M. haemobos” identified in the present study (BovCMhbos61 and BufCMhbos01) branched with previous reported isolates from China, Japan, Germany and Switzerland, which formed a separate cluster from *M. wenyonii* group, together with *M. haemocanis* and *M. haemofelis* as previously described [14].

## Discussion

To our knowledge, the present study provides the first molecular evidence for the occurrence of hemotropic mycoplasmas infections in dairy cattle and buffalo in Cuba, and throughout the Caribbean region. A relatively high prevalence of bovine hemoplasma infections was detected by real-time PCR in the studied buffalo and dairy cattle herds. The infection with *M. wenyonii* was predominant in both host species, whereas “*Ca.* M. haemobos” was detected with a lower frequency, and always as a co-infection with *M. wenyonii*. Hofmann-Lehmann et al. [7] were the first to report hemoplasma infections in cattle that underwent concurrent infections with other vector-borne pathogens, during an outbreak of fatal hemolytic anemia in a Swiss cattle herd. In a follow-up study, Meli et al. [14] described in Swiss cattle a higher prevalence of *M. wenyonii* (63.5%) compared to “*Ca.* M. haemobos” (49.6%) and 42.9% of co-infections.

Early studies in Japanese cattle by Tagawa et al. [6] reported lower prevalences (PCR-positive) at 21.8 and 16.7% of the animals for *M. wenyonii* and “*Ca.* M. haemobos”, respectively, whereas 5.1% of these were infected with both hemoplasma species. Later, in an epidemiological survey carried out in different regions of Japan, Tagawa et al. [20] reported that the prevalence rates were 38.5% for *M. wenyonii* and 39.1% for “*Ca.* M. haemobos”, with an overall prevalence of 64.7% for all bovine hemoplasma infections. A high infection rate of “*Ca.* M. haemobos” (41.7%) has also been reported in cattle and buffalo from tropical China [15]. In Brazil, epidemiological surveys performed by Girotto et al. [16] and Witter et al. [21] reported prevalence rates for “*Ca.* M. haemobos” in dairy cattle of 60.9 and 64.2%, respectively. Hence, our findings suggest that these pathogens are widespread in cattle and buffalo herds in Cuba, with similar patterns to other regions around the world.

Although the geographical difference in the bovine hemoplasma species infection rates has not been thoroughly investigated, it may be related to various factors including the activity of arthropod vectors for hemoplasma transmission, the age and sex of the bovine species, as well as the animal husbandry practice. In Cuba, livestock is maintained under three major management practices: intensive, semi-intensive and extensive systems. In the sampled farms dairy cattle herds were maintained under a semi-intensive management system, while buffalo herds were reared solely under an extensive system. Ybanez et al. [22] described that pastured livestock have a higher risk of exposure to blood-sucking arthropods that are capable of transmitting hemoplasma infections compared to stabled animals. So far, the epidemiological data on *M. wenyonii* and “*Ca.* M. haemobos” in the Caribbean region are scarce or inexistent, with only a few studies in Cuba conducted almost 30 years ago. A similar situation is found in the Americas mainland, whereby some studies conducted in northern and southern Brazil have reported similar infection rates as in Europe and Asia [16, 21, 23]. As such, further investigation is necessary to document prevalence rates in cattle and buffalo herds in Cuba and across the Caribbean.

In the present study, the average PCV values of the studied animals were comparable to those described for healthy dairy cattle and buffalo. A slightly decrease in the PCV values according to the reference values for each host species was observed in some hemoplasma-infected (one cattle and four buffalo) and non-infected (four cattle and one buffalo) animals. However, the hemoplasma-infected dairy cattle and buffalo did not appear to be clinically affected and no significant association was observed between the hemoplasma PCR-positivity and the occurrence of anemia. The decrease in the PCV values derives from anemia, which might be in younger animals due to the elimination of the fetal erythrocytes that can cause significant reductions in the levels of these blood cells in animals with ages between three and four months old [24]. Besides this, it has been observed that dairy cattle and buffalo can present variations in the PCV levels depending on age, breeding region, nutritional and sanitary management [25]. As none of the infected dairy cattle or buffalo showed clinical manifestations of severe anemia, hematological parameters were not subjected to in-depth investigation.

In the present study, dairy cattle and buffalo that were one-year-old and older showed a higher risk of bovine hemoplasma infection than those less than one-year-old. In agreement with our findings, Tagawa et al. [20] reported a higher prevalence of *M. wenyonii* infections in Japanese cattle from one to three years of age compared with younger animals. In addition, Congli et al. [26] reported in China that cattle from two to four years of age were most susceptible to *M. wenyonii* infections compared with younger and older animals. A similar observation was reported by Girotto et al. [16], who detected in southern Brazil that female cattle above two-years-old presented a higher prevalence rate of “*Ca.* M. haemobos” infections. These results are consistent with the fact that most two-year-old dairy cattle and buffalo that have been put out to pasture stay longer in the herd and have experienced their first pregnancy and delivery. The increased risk of contact with potential vectors in the pasture, as well as pregnancy-induced immune depression, may have contributed to the higher prevalence of observed infections [20].

In the present study, both domestic bovine species were found infested with *R. microplus* tick species, although a higher prevalence and infestation level was found in cattle than in buffalo. Sajid et al. [27] described that the higher host susceptibility for tick infestation found in cattle than in buffalo is due to the thinner skin and dry habitat of the cattle as compared to thicker skin and swampy habitats of the buffalo. In the present study, a surprising result was that a lack of tick infestation in buffalo was associated with a higher prevalence of “*Ca.* M. haemobos” infection; it was noted that only younger buffalo were found tick-infested. Reasons for higher infestation in younger animals include (i) less developed immune system that has yet to be challenged by exposure to tick infestation and (ii) softer skin and tissue, facilitating easy penetration of mouth parts of ticks into the body of the host [28]. Mohd Hasan et al. [29] reported the first molecular evidence of the presence of *M. wenyonii* in *R. microplus* ticks collected from Malaysian cattle. In addition, Hornok et al. [30] and Song et al. [9] reported the potential role of blood-sucking flies, lice and mosquitoes as mechanical vectors of bovine hemoplasmas, as well as the transplacental transmission mode.

Considering that the only tick species identified in the present study infesting dairy cattle and buffalo was *R. microplus*, which is widely distributed in the Cuban mainland, and the resistance of buffalo to tick infestation, we think that other vectors or transmission modes could be involved in the occurrence of *M. wenyonii* and “*Ca.* M. haemobos” in the studied region; however, this observation needs further investigation.

## Conclusions

The present study constitutes the first molecular evidence of *M. wenyonii* and “*Ca.* M. haemobos” infections in Cuban dairy cattle and buffalo. Our results indicate a wide distribution of bovine hemoplasma infections among dairy cattle and buffalo herds in the studied region. No obvious anemia was observed in infected animals; however, their infection stage (acute infection *versus* carrier) was unknown. Infected animals probably remain chronic carriers. Our study provides new information on the biodiversity of vector-borne pathogens in dairy cattle and buffalo population in Cuba. However, it is impossible to evaluate the infection rate epidemiologically, because a limited number of samples were investigated. Further studies are required to clarify the pathogenicity and epidemiology, as well as to assess the impact that these two bovine hemoplasmas have on the livestock industry in Cuba.

## Methods

### Aim and design of the study

The aim of this study was to investigate for the first time the presence of bovine hemoplasma species in cattle and buffalo from Cuba using molecular techniques. For this purpose, blood samples were collected from two cattle (*Bos taurus*) and two water buffalo (*Bubalus bubalis*) herds.

### Collection of blood samples and DNA extraction

A total of 80 field blood samples were collected on four farms in the Mayabeque Province of Cuba from March to May, 2014 (Fig. 1). The studied farms were small, low-income and family-owned operations that functioned as dairy farms.

For all the animals, blood samples were collected by jugular venopuncture using 10 ml vacutainer collecting tubes containing EDTA (BD Vacutainer®, Becton Dickinson Vacutainer Systems, Franklin Lakes, NJ, USA). The hematocrit was assessed by microcentrifugation from each blood sample in a Jouan Hema-C microhematocrit centrifuge (Hawksley and Sons, Ltd, Sussex, UK; 18,600× *g*, 5 min); the value was determined with a DAMON/IEC hematocrit reader (Damon/IEC Division, Needham Heights, MA, USA). The remaining EDTA blood samples were stored at -20 °C prior to nucleic acid extraction. TNA was extracted from 100 μl of blood using a DNeasy Blood and Tissue Kit (Qiagen, Valencia, CA, USA) according to the manufacturer’s instructions. The concentration and purity of the TNA was determined by measuring absorbance at 260 and 230 nm using a Colibri Microvolume Spectrophotometer (Titertek-Berthold, Pforzheim, Germany), and TNA was stored at -20 °C until use for qPCR analysis.

### Collection of tick samples from cattle and buffalo

An average of ten adult tick specimens were collected from different body parts (ears, head, neck, pectoral, inguinal and tail) of each infested animal, and stored in polypropylene tubes containing isopropyl alcohol. Ticks collected from cattle and buffalo were taxonomic identified to the species level based on the dichotomous key of Barros-Battesti et al. [31]. In the parasitized animals, the tick infestation level was measured subjectively based on the observation of adult tick specimens on the cattle and buffalo, and sorted into two infestation levels (absent/low or moderate/high) according to Labruna et al. [32]. All ticks included in the study were engorged ticks collected directly from infested cattle and buffalo. Therefore, the ticks were not analyzed for the presence of bovine hemoplasmas, since the presence of hemoplasmas in engorged ticks may have resulted from feeding on the animals and cannot directly be related to a vector potential of the ticks.

### Real-time PCR assays

The presence of *M. wenyonii* and “*Ca.* M. haemobos” DNA was detected using species-specific real-time TaqMan PCR assays based on *16S* rRNA genes previously described by Meli et al. [14]. The presence of amplifiable DNA and the absence of PCR inhibitors were confirmed by the successful amplification of the bovine glyceraldehyde-3-phosphate dehydrogenase gene (GAPDH) on all samples [33]. The primers and probes were custom-synthesized at a commercial source (Microsynth, Balgach, Switzerland) and partly modified from the original publications (Table 4). For real-time quantitative assays, 5 μl of the extracted genomic DNA template was combined with 900 nM of each oligonucleotide primer and 250 nM of the corresponding probe in a total reaction volume of 20 μl, using 10 μl of 2× TaqMan Universal PCR Master Mix (Thermo Fisher Scientific, Reinach, Switzerland),) and 0.25 μl (0.5 U) of uracyl DNA N-glycosylase (Roche Diagnostics, Mannheim, Germany) per reaction. TaqMan PCR reactions were mixed in 96-well optical plates (Applied Biosystems, Thermo Fisher Scientific). The PCR samples were subjected to 45 cycles of amplification in an ABI 7500 Fast Real-Time PCR System (Applied Biosystems, Thermo Fisher Scientific) under the following conditions: 50 °C for 2 min (uracil N-deglycosylase digest), 95 °C for 10 min (AmpliTaq Gold pre-activation), and then 45 cycles of 95 °C for 15 s and 60 °C for 1 min. The ABI 7500 Fast Real-Time PCR system provided a cycle by cycle measurement of the fluorescence emission from each reaction. For each batch of samples, a negative control (RNase-free water) and positive controls (*M. wenyonii* and “*Ca.* M. haemobos” synthetic DNA, GeneArt String DNA; Thermo Fisher Scientific) were included.

**Table 4:** Primers and probes used in this study for the real-time TaqMan PCR assays, and sequence analysis of *M. wenyonii* and “*Ca*. M. haemobos” from dairy cattle and buffalo

Target	Primer/Probe	Sequence (5'-3')	Amplicon length (bp)	Reference
Bovine GAPDH	GAPDH.463f	GGCGTGAACCACGAGAAGTATAA	120	Modified from Leutenegger et al. [33]
	GAPDH.582r	CCCTCCACGATGCCAAAGT		
	GAPDH.489p	ATTO550-AYACCCTCAAGATTGTCAGCAATGCCTCCT-BHQ-2		
*M. wenyonii*	MwenyoniiF	CCACGTGAACGATGAAGGTCTT	119	Modified from Meli et al. [14]
	MwenyoniiR	GGCACATAGTTAGCTGTCACTTATTCAA		
	Mwen_P	6-FAM-AGTACCATCAAGGCGYGCTCATTTCCTAG-BHQ-1		
	MycWen15f^a^	ACACATGCAAGTCGAACGAG	1360	This study
	MycWen1374r^a^	ATTGAATGTGGTTTTGACTAGTACTTT		
“*Ca.* M. haemobos”	Mwen_short.forw	CCATGTGAACGATGAAGGTCTTT	90	Modified from Meli et al. [14]
	Mwen_short.rev	AGTTTGCTGTCACTTATTCATGAGGTA		
	Mwen_short.p	YY-CT**A**^1^TC**A**^1^GTTRTT**A**^1^TCCCTC**A**^1^TAA-BHQ-1		
	MHBforw^a^	GAATTAATGCTGATGGTATGCCTAA	1393	Meli et al. [14]
	MHBrev^a^	CCAATCAGAATGTTCACTCTAGATGC		

^a^Primers used for the sequencing reactions

*Abbreviations*: BHQ, black hole quencher; 6-FAM, 6-carboxyfluorescein; YY, Yakima yellow; **A**^1^, 2-aminopurin: internal modification to increase melting temperature in substitution of the minor groove binder (used in the original publication)

### Sequencing and phylogenetic analysis of the *16S* rRNA gene

For sequence analysis, the near full-length *16S* rRNA genes of *M. wenyonii-* and “*Ca.* M. haemobos”-positive samples were amplified using the primer sets shown in Table 4. The samples for sequencing were randomly chosen among the PCR-positive samples. For both pathogens, 5 μl of extracted TNA were included in a total volume of 50 μl containing 10 μl of 5× Phusion HF buffer (Finnzymes, Espoo, Finland), 500 nM each primer, 200 nM each deoxynucleoside triphosphate (dNTP) (Sigma-Aldrich, Buchs, Switzerland), and 1 U Phusion DNA Polymerase (Finnzymes). Amplification was performed using a T-personal 48 thermal cycler (Biometra GmbH, Göttingen, Germany) under the following conditions: an initial denaturation step at 98 °C for 3 min; 35 cycles of 98 °C for 10 s, 60 °C for 30 s and 72 °C for 30 s; and a final extension step at 72 °C for 10 min. PCR products were subjected to electrophoresis in a 1.5% agarose gel (100V, 40 min), pre-stained with gel red and visualized under ultraviolet light. PCR products were purified with the QIAquick Gel Extraction Kit (Qiagen) according to the manufacturer’s instructions, and submitted for direct sequencing at a commercial lab (Microsynth, Balgach, Switzerland). Sequences were identified by checking the specified sequence against existing sequences using the BLASTn search program (http://www.ncbi.nlm.nih.gov/blast/Blast.cgi).

For phylogenetic and molecular evolutionary analysis, the sequences were aligned with known hemoplasma sequences from GenBank using ClustalW [34] and manually adjusted when necessary. Only nucleotides that were available for all included sequences were used in the phylogenetic analysis. A bootstrap phylogenetic tree was inferred to determinate the relationship of the detected bovine species to know hemoplasma species. The tree was created by the neighbor-joining method [35] using a distance matrix corrected for nucleotide substitutions based on the Tamura 3-parameter model. The data set was resampled 1000 times to generate bootstrap values. Phylogenetic and molecular evolutionary analyses were conducted using MEGA v.7.0.14 [36].

### Statistical analysis

The association between a PCR-positive samples to *M. wenyonii* and “*Ca.* M. haemobos” and variables such as age, presence of ticks and anemia, were analyzed using Fisher’s exact test. The prevalence rates were calculated with 95% confidence intervals (CI). PCV was analyzed using references values described for each bovine species, and was used as parameter of anemia for dairy cattle (PCV < 24% indicates anemia, reference range: 24–46%) [37] and buffalo (PCV < 30% indicates anemia, reference range: 30–50%) [38]. The age of the animals was also analyzed as a categorical variable with animals classified as ≤ 1-year-old and > 1 and < 3 years-old. None of the studied animals was older than 3 years old. In addition, quantitative variables, such as age and hematocrit, were analyzed by the Mann-Whitney U-test. Statistical analyses were performed using the software Jamovi 0.8.1.10 (Jamovi project, 2017). Differences were considered statistically as significant if *P* < 0.05.

### Nucleotide sequence accession numbers

The nucleotide sequences have been submitted to the GenBank database under the accession numbers MG948624, MG948626, MG948628 and MG948631.

## Abbreviations

16S rRNA: 16 Svedberg ribosomal ribonucleic acid
6-FAM: 6-carboxyfluorescein
A*: 2-aminopurin
BHQ: black hole quencher
CI: confidence interval
GAPDH: glyceraldehyde-3-phosphate dehydrogenase gene
PR: prevalence ratio
YY: Yakima yellow
